# Combined 5‐aminolevulinic acid and ferric ammonium citrate treatment promotes hair follicle growth by activating dermal papilla cells

**DOI:** 10.1002/2211-5463.70230

**Published:** 2026-03-24

**Authors:** Han‐Wook Ryu, Eok‐Soo Oh, Sewoon Kim

**Affiliations:** ^1^ R&D center, Easytem Co., Ltd. Seoul Republic of Korea; ^2^ Department of Life Sciences Ewha Womans University Seoul Republic of Korea

**Keywords:** 5‐aminolevulinic acid, dermal papilla cell, ERK and AKT signaling, hair follicle, hair loss

## Abstract

Although 5‐aminolevulinic acid (5‐ALA) has shown potential for hair growth in previous studies, the molecular mechanisms remain unclear. In this study, we investigated the combined effect of 5‐ALA with ferric ammonium citrate (FAC) in human hair follicle models. At low micromolar concentrations, 5‐ALA/FAC treatment increased proliferation of dermal papilla cells, keratinocytes, fibroblasts, and adipose‐derived stem cells, and rapidly activated ERK and AKT signaling. This treatment also upregulated hair‐inductive genes and restored their suppression by dihydrotestosterone or oxidative stress. In functional assays, 5‐ALA/FAC treatment induced hair follicle‐like structures in reconstituted skin and enhanced hair shaft elongation in *ex vivo* organ culture. These findings indicate that 5‐ALA/FAC treatment stimulates human hair follicle growth and support its potential as a therapeutic candidate for hair loss.

## Graphical abstract

5‐Aminolevulinic acid combined with ferric ammonium citrate (5‐ALA/FAC) stimulates dermal papilla cell activity and promotes hair follicle growth. The treatment enhances ERK and AKT signaling, increases hair‐inductive gene expression, and restores dermal papilla function suppressed by dihydrotestosterone and oxidative stress, resulting in enhanced hair follicle formation and hair shaft elongation.

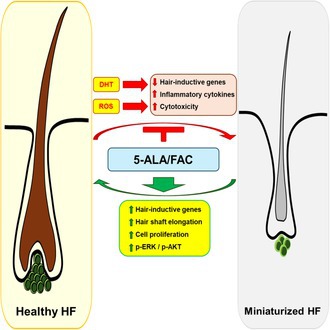

**Abbreviations**
5‐ALA: 5‐aminolevulinic acid
FAC: ferric ammonium citrate
DPCs: dermal papilla cells
iDPCs: immortalized dermal papilla cells
HFSCs: epithelial hair follicle stem cells
DHT: dihydrotestosterone
ROS: reactive oxygen species
PpIX: protoporphyrin IX
HO‐1: heme oxygenase‐1
qRT‐PCR: quantitative real‐time polymerase chain reaction
SD: standard deviation
SE: standard error

The hair follicle undergoes a cyclic process consisting of growth (anagen), regression (catagen), and resting (telogen) phases. Transition from telogen to anagen is a highly regulated event that determines the onset of new hair shaft formation. Dermal papilla cells (DPCs), specialized mesenchymal cells at the base of hair follicles, serve as a signaling center that coordinate communication with epithelial hair follicle stem cells (HFSCs). Through secretion of paracrine growth factors, DPCs play a pivotal role in triggering telogen to anagen transition, promoting proliferation and differentiation of keratinocytes, and supporting subsequent hair shaft elongation. Thus, activation of DPCs is a key determinant of anagen induction and hair cycle progression [1, 2].

Hair loss arises when the hair cycle is disrupted by genetic, hormonal, and environmental factors. Among these, dihydrotestosterone (DHT) is a critical mediator that impairs DPC function, suppresses expression of hair‐inductive genes, and shortens anagen duration, thereby contributing to androgenetic alopecia [3, 4]. In addition, oxidative stress induced by reactive oxygen species (ROS) has been implicated in DPC senescence and apoptosis, leading to hair follicle miniaturization [5, 6]. Other contributing factors include inflammatory cytokines and microvascular insufficiency, all of which converge on impaired DPC activity and disturbed hair follicle homeostasis [7, 8, 9]. Although pharmacologic options such as minoxidil, 5‐α‐reductase inhibitors for androgenetic alopecia and JAK inhibitors for severe alopecia areata can restore hair‐growth signaling, their use is limited by adverse effects (scalp irritation/hypertrichosis with minoxidil; sexual adverse events with finasteride; infections with systemic JAK inhibitors) [10, 11, 12]. These considerations underscore the need for well‐tolerated natural therapeutics that can safely reinforce anagen‐promoting pathways.

5‐Aminolevulinic acid (5‐ALA) is an endogenous precursor in the heme biosynthetic pathway. 5‐ALA is converted to protoporphyrin IX (PpIX), which preferentially accumulates in rapidly proliferating cells and generates cytotoxic reactive oxygen species upon visible‐light activation [13]. Clinically, 5‐ALA photodynamic therapy (ALA‐PDT) has been widely adopted in oncology, particularly for skin cancers, for photodynamic diagnosis and fluorescence‐guided resection by exploiting selective protoporphyrin IX accumulation [14, 15]. ALA‐PDT has also demonstrated therapeutic benefit in severe acne, highlighting its translational potential from medical to cosmetic dermatology [16].

Over the past two decades, ALA‐PDT has been evaluated in alopecia areata with inconsistent outcomes [17]. Some studies reported no regrowth, whereas others described partial or complete regrowth in subsets of patients. Independent of light activation, topical 5‐ALA with an iron source has promoted hair growth in mice [18]. However, these observations have not clarified how 5‐ALA might promote hair growth in human systems. Here, we investigate 5‐ALA combined with ferric ammonium citrate (FAC) in human cells and reconstituted tissue models, and we examine mechanisms linked to the telogen to anagen transition. This study aimed to provide mechanistic evidence for 5‐ALA as a well‐tolerated therapeutic candidate for hair loss.

## Materials and methods

### Cell culture

Human keratinocytes (HaCaT; kindly provided by Prof. Tae‐Yoon Kim, The Catholic University of Korea), immortalized dermal papilla cells (KNU201; iDPCs, Epibiotech, Incheon, Republic of Korea), adipose‐derived stem cells (STC004; hADSCs, Epibiotech), and normal human dermal fibroblasts (CC‐2511; NHDF‐Ads, Lonza) were maintained in their respective growth media under standard conditions (37 °C, 5% CO_2_). HaCaT and iDPCs were cultured in DMEM (Welgene, Gyeongsan, Republic of Korea) containing 10% fetal bovine serum (FBS) and antibiotics (100 U·mL^−1^ penicillin, 100 μg·mL^−1^ streptomycin). hADSCs were cultured in α‐MEM supplemented with 10% FBS and 1% antibiotics, and NHDF‐Ads in EGM‐2 (Lonza, Basel, Switzerland) containing 2% FBS and supplied growth factors. hADSCs and NHDF‐Ads were passaged at 70–90% confluence using trypsin–EDTA and used between Passages 4 and 8.

Commercially obtained cell lines were supplied with authentication documentation from the vendors. All commercially obtained cell lines were purchased within the past 3 years. No additional authentication beyond supplier verification was performed in our laboratory. All cell cultures were maintained under sterile conditions and were routinely monitored during culture. No evidence of mycoplasma contamination was observed.

### Preparation of 5‐ALA/FAC mixture

5‐Aminolevulinic acid phosphate was kindly provided by KIYAN PHARMA (Tokyo, Japan), and ferric ammonium citrate was purchased from SAMCHUN CHEMICALS (Seoul, Republic of Korea). Both reagents were dissolved in sterile water and mixed at an equimolar ratio (1 : 1) to prepare a 100 mm stock solution.

### Cell viability assay

Cells were seeded in 96‐well plates (1 × 10^3^ cells/well) and treated for 48 h with 5‐ALA/FAC (1 nm–1 μm). Viability was measured using the EZ‐Cytox reagent (Dogenbio, Seoul, Republic of Korea) according to the manufacturer's instructions. Absorbance was read at 450 nm, and viability was expressed as:

$$\text{Cell viability}\;\left(\%\right)=\left(\frac{\text{Sample}-\text{Backgound}\;}{\text{Control}-\text{Backgound}\;}\right)\times 100$$

### Quantitative real time‐PCR

Total RNA was extracted using the Quick‐RNA Mini Prep Kit (Zymo Research, Irvine, CA, USA), and cDNA was synthesized from 1 μg RNA with ProtoScript® First Strand cDNA Synthesis Kit (NEB, Ipswich, MA, USA). qRT‐PCR was performed using TOPreal™ SYBR Green PreMix (Enzynomics, Daejeon, Republic of Korea) on a QuantStudio™ 5 system (Applied Biosystems, Foster City, CA, USA). mRNA levels of *VCAN*, *SOX2*, *FGF2*, *IL6*, and *TGFB1* were normalized to *GAPDH*. The primer sequences (5′–3′) used in this study were as follows: *VCAN* forward, CAGTGGAATGATGTTCCCTGC; *VCAN* reverse, CGGATAGTTGGAAGGTGACGT; *SOX2* forward, CTTTTGTCGGAGACGGAGAAG; *SOX2* reverse, CCTTCTTCATGAGCGTCTTGG; *FGF2* forward, GCTAACCGTTACCTGGCTATG; *FGF2* reverse, CTGGTGTATTTCCTTGACCGG; *IL6* forward, CAGAGCTGTGCAGATGAGTAC; *IL6* reverse, CTGGCATTTGTGGTTGGGTCA; *TGFB1* forward, TACATTTGGAGCCTGGACACG; *TGFB1* reverse, GATCATGTTGGACAGCTGCTC; *GAPDH* forward, CACTGCCACCCAGAAGACTG; and *GAPDH* reverse, GTGAGCTTCCCGTTCAGCTC.

### Immunoblot analysis

Cells were lysed in RIPA buffer containing protease and phosphatase inhibitors (Thermo Fisher Scientific, Waltham, MA, USA). Equal protein amounts were separated by SDS/PAGE and transferred to PVDF membranes. Membranes were probed with the following primary antibodies: AKT (#9272S; Cell Signaling Technology, Danvers, MA, USA), ERK1/2 (#9102S; Cell Signaling Technology), phospho‐AKT (#4060S, Ser473; Cell Signaling Technology), phospho‐ERK1/2 (#4370S, Thr202/Tyr204; Cell Signaling Technology), and β‐actin (sc‐47778; Santa Cruz Biotechnology, Dallas, TX, USA). HRP‐conjugated secondary antibodies were applied, and immunoreactive bands were visualized using a chemiluminescence detection system (Bio‐Rad, Hercules, CA, USA). Phospho‐kinase profiling was performed using the Proteome Profiler Human Phospho‐Kinase Array Kit (R&D Systems, Minneapolis, MN, USA) according to the manufacturer's instructions. iDPCs were treated with 1 μm 5‐ALA/FAC for 30 min and harvested for protein extraction. The relative levels of phosphorylated kinases were quantified using the imagej software by measuring signal intensities from duplicate spots of the control and 5‐ALA/FAC‐treated samples.

### 3D hair follicle‐like structure formation

Neoderm®‐DF (Tego Science, Seoul, Republic of Korea) was placed in six‐well plates containing 3.5 mL maintenance medium and treated with either 100 μm minoxidil or 0.6% 5‐ALA/FAC for 7 days. Media were refreshed every 3 days. Samples were fixed, paraffin‐embedded, and sectioned at 5 μm thickness. Sections were stained with hematoxylin–eosin or subjected to immunofluorescence staining using anti‐versican (MA5‐27638; Invitrogen, Carlsbad, CA, USA) or anti‐pan‐keratin (NBP3‐07280; Novus Biologicals, Centennial, CO, USA) primary antibodies. Fluorescein (FITC) AffiniPure® Goat Anti‐Mouse IgG (115‐095‐003; Jackson ImmunoResearch Laboratories, West Grove, PA, USA) or Rhodamine Red™‐X (RRX) AffiniPure® Goat Anti‐Rabbit IgG (111‐295‐003; Jackson ImmunoResearch Laboratories) secondary antibodies were used, followed by DAPI counterstaining. Images were captured by fluorescence microscopy.

### Human hair organ culture

Human hair follicles were obtained from the New Hair Institute (Seoul, Republic of Korea) with the understanding and written informed consent of each donor. All procedures conformed to the principles outlined in the Declaration of Helsinki. The study protocol was approved by the Institutional Review Board of Epibiotech (IRB No. 70094321‐202307‐BR‐003‐01). Microdissected follicles were cultured in serum‐free Williams' E medium containing L‐glutamine (2 mm), insulin (10 μg·mL^−1^), hydrocortisone (10 ng·mL^−1^), and antibiotics. Treatment medium with 1 μm 5‐ALA/FAC was replaced every 2 days. Hair shaft elongation was measured on Day 5 from calibrated images (*n* = 20 follicles).

### Statistical analysis

Data from cell viability assays and quantitative real‐time PCR (qPCR) experiments are presented as mean ± standard deviation (SD) and were obtained from technical triplicates. Hair length measurements are presented as mean ± standard error (SE), with a sample size of *n* = 20 hairs per group. Statistical analyses were performed using Microsoft Excel. Comparisons between two groups were conducted using a two‐tailed unpaired Student's *t*‐test. A *p*‐value < 0.05 was considered statistically significant.

## Results

### 5‐ALA/FAC promotes human cell proliferation

**Fig. 1:**
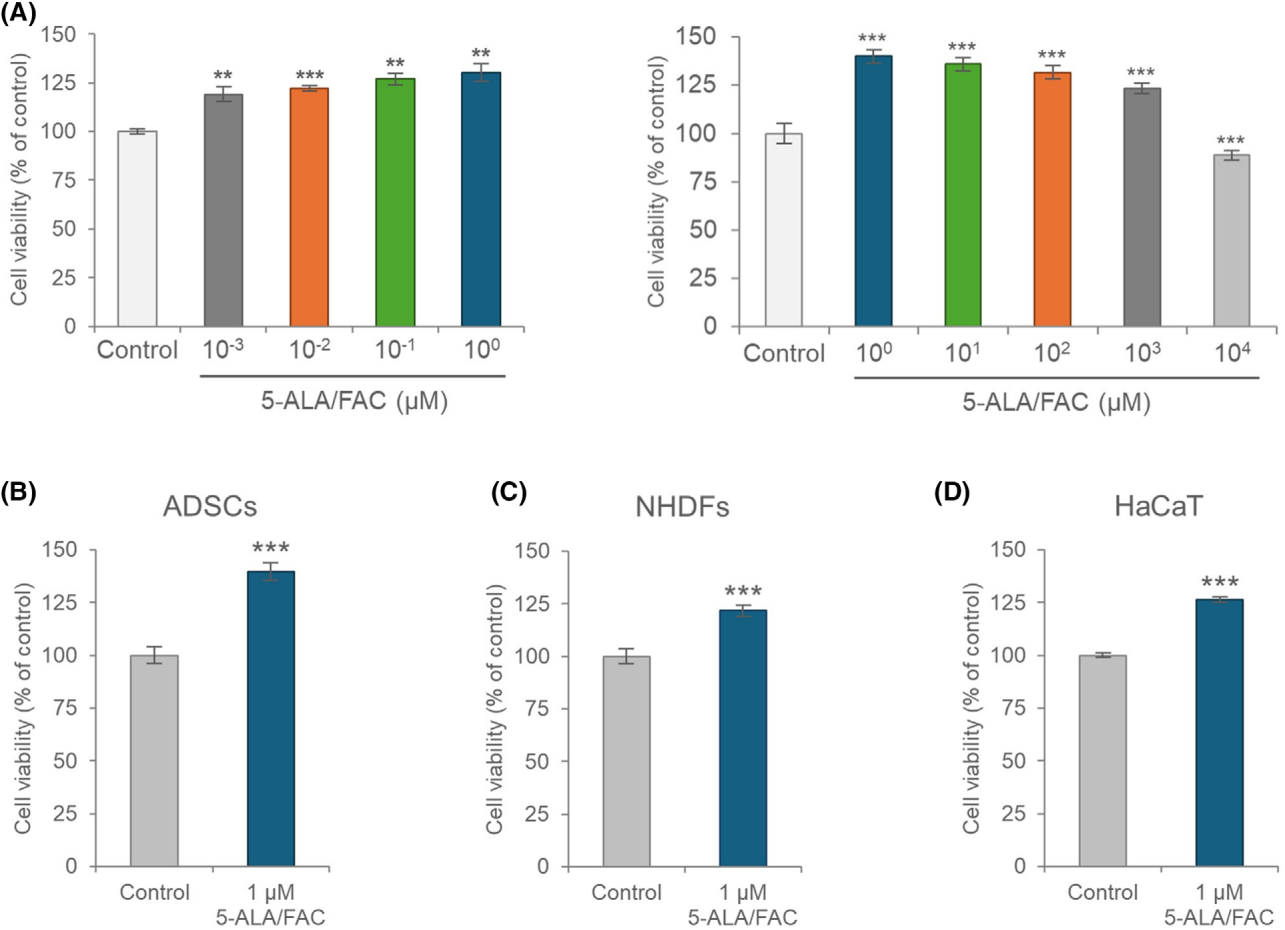
5‐ALA/FAC enhances proliferation of human hair‐related cells. (A) Cell viability of immortalized human dermal papilla cells (iDPCs) treated with increasing concentrations of 5‐aminolevulinic acid combined with ferric ammonium citrate (5‐ALA/FAC; 10^−3^–10^0^ μm, left) or 5‐ALA/FAC at higher doses (10^0^–10^4^ μm, right) for 48 h. (B–D) Viability of (B) human adipose‐derived stem cells (ADSCs), (C) normal human dermal fibroblasts (NHDFs), and (D) HaCaT keratinocytes after 1 μm 5‐ALA/FAC treatment for 48 h. Cell viability was determined using a water‐soluble tetrazolium salt‐1 (WST‐1, EZ‐Cytox) assay and is expressed as a percentage of control (mean ± SD, *n* = 3). Control samples were treated with vehicle only. *P < 0.05, **P < 0.01, ***P < 0.001 vs. vehicle control. Statistical analysis was performed using a two‐tailed unpaired Student's *t*‐test.

To assess the effect of 5‐ALA/FAC on the proliferation of hair‐related cells, WST‐1 assays were performed. In immortalized human dermal papilla cells (iDPCs), treatment with 5‐ALA/FAC resulted in the highest cell viability at 1 μm, which was significantly greater than the untreated control (*p* < 0.01). Cell viability declined at concentrations either lower or higher than 1 μm, and marked cytotoxicity was observed at 10 mm (Fig. 1A). Similarly, treatment with 1 μm 5‐ALA/FAC significantly enhanced viability in adipose‐derived stem cells (ADSCs), normal human dermal fibroblasts (NHDFs), and HaCaT keratinocytes (Fig. 1B–D). These findings suggest that 5‐ALA/FAC promotes the proliferation of multiple hair‐related cell types, with an optimal effect at 1 μm.

### 5‐ALA/FAC promotes functional activity of iDPCs

**Fig. 2:**
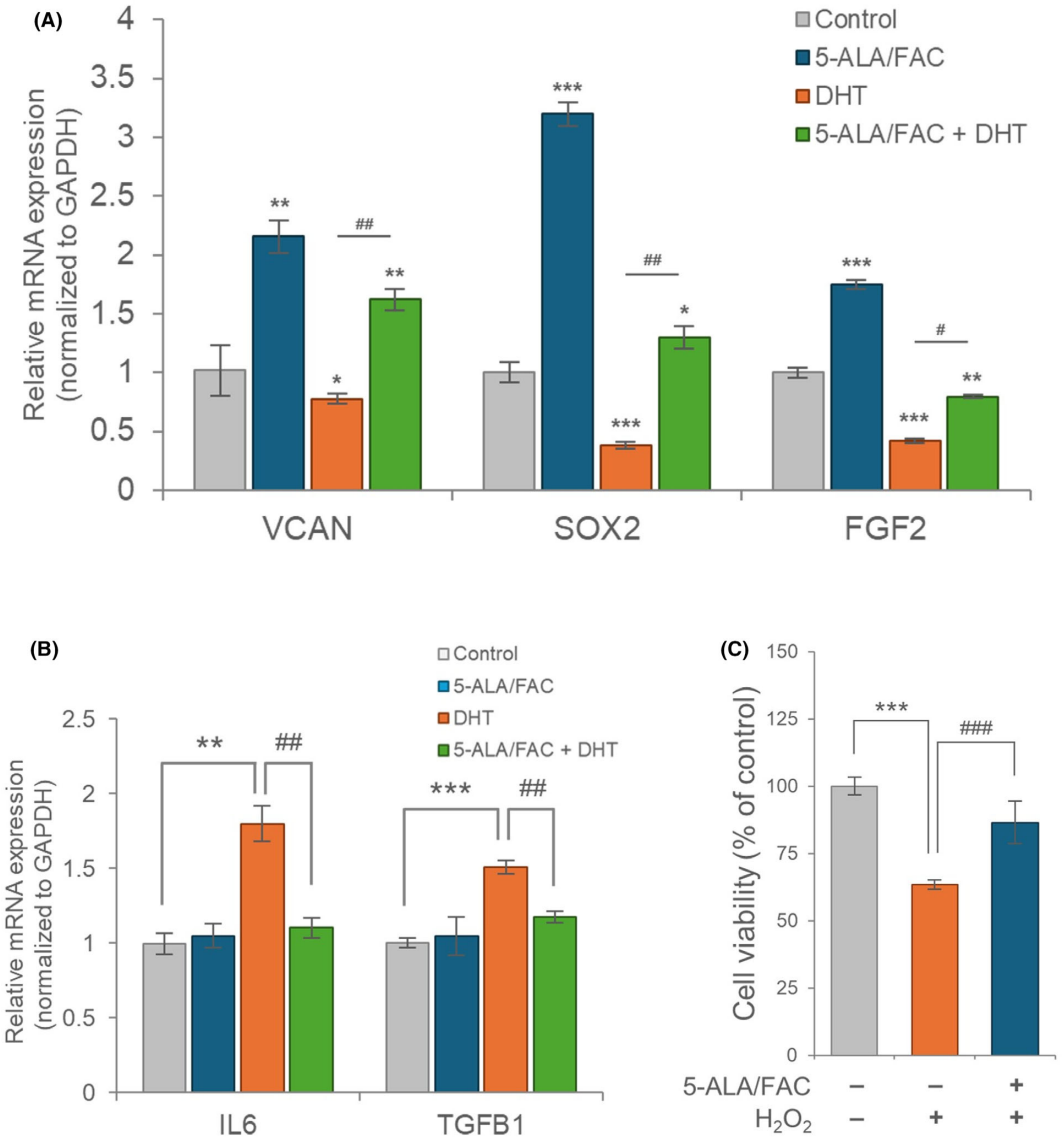
5‐ALA/FAC restores hair‐inductive gene expression suppressed by DHT and protects iDPCs from oxidative stress. (A) Relative mRNA expression of *VCAN*, *SOX2*, and *FGF2* in iDPCs treated with 1 μm 5‐ALA/FAC and/or 100 nm 5α‐dihydrotestosterone (DHT) for 8 h, analyzed by qRT‐PCR and normalized to *GAPDH*. (B) Expression of *IL6* and *TGFB1* under the same treatment conditions. (C) Protective effects of 5‐ALA/FAC against H₂O₂‐induced cytotoxicity. iDPCs were pretreated with 400 μm H_2_O_2_ for 4 h, followed by incubation with 1 μm 5‐ALA/FAC for 24 h. Cell viability was assessed by WST‐1 assay. Control samples were treated with vehicle only. Data are mean ± SD (*n* = 3). *P < 0.05, **P < 0.01, ***P < 0.001 vs. vehicle control; ^#^ *P* < 0.05, ^##^ *P* < 0.01, ^###^ *P* < 0.001 vs. DHT‐ or H_2_O_2_‐treated group. Statistical analysis was performed using a two‐tailed unpaired Student's *t*‐test.

DPCs are essential for hair follicle development and hair cycle regulation. Activation of DPCs can induce telogen to anagen transition followed by inducing hair shaft elongation [2]. Therefore, we examined whether 5‐ALA/FAC promotes the expression of hair‐inductive genes featured by DPCs activation. 5‐ALA/FAC elevated the mRNA expression of *VCAN*, *SOX2*, and *FGF2* (Fig. 2A). DHT causes DPCs dysfunction through attenuating hair‐inductive genes expression [3], activating paracrine signaling including IL‐6 and TGF beta [7, 8], and ROS generation [5]. We investigated protective effects of 5‐ALA/FAC against attenuating DPC activity induced by DHT treatment. In the presence of DHT, hair‐inductive genes were downregulated, and these effects were restored upon treatment with 5‐ALA/FAC together (Fig. 2A). In addition, 5‐ALA/FAC inhibited to enhance the expression of *IL6* and *TGFB1* by DHT treatment (Fig. 2B). A protective activity of 5‐ALA/FAC from ROS were evaluated at the sublethal concentration of H_2_O_2_. The reduced cell viability of iDPCs by H_2_O_2_ treatment was significantly rescued by 5‐ALA/FAC (Fig. 2C). These results showed that 5‐ALA/FAC not only promoted anagen‐specific gene expression in DPCs in the normal growth condition but also restored the DPCs dysfunctions induced by DHT and oxidative stress.

### 5‐ALA/FAC activates ERK and AKT independently of HO‐1 induction

**Fig. 3:**
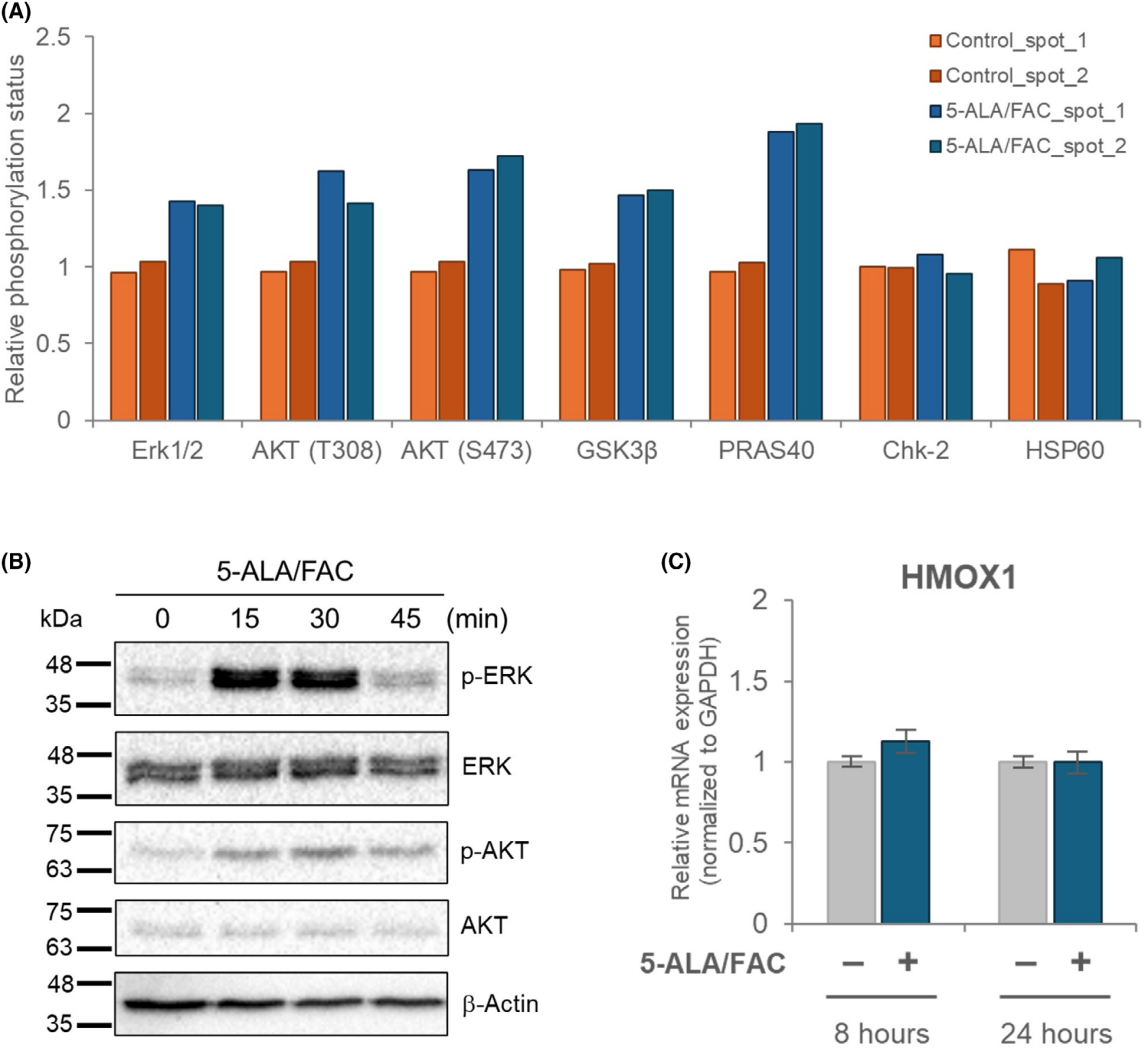
5‐ALA/FAC activates ERK and AKT signaling independently of HO‐1 induction in iDPCs. (A) Phospho‐kinase array analysis showing increased phosphorylation of ERK1/2, AKT (T308 and S473), GSK3β, and PRAS40 after 1 μm 5‐ALA/FAC treatment. (B) Representative immunoblots showing p‐ERK and p‐AKT activation in iDPCs treated with 1 μm 5‐ALA/FAC for 0–45 min. (C) *HMOX1* mRNA levels after 8 and 24 h of 5‐ALA/FAC treatment analyzed by qRT‐PCR and normalized to *GAPDH*. Data are presented as mean ± SD (*n* = 3). Control samples were treated with vehicle only.

To elucidate the mechanisms underlying 5‐ALA/FAC‐induced activation of dermal papilla cells, we first performed a phospho‐kinase array. This analysis revealed increased phosphorylation of ERK1/2 and AKT in iDPCs treated with 5‐ALA/FAC. Consistent with the enhanced AKT activation, phosphorylation of its downstream substrates, GSK3β and PRAS40, was also upregulated (Fig. 3A). To validate these findings, we examined the phosphorylation status of ERK and AKT by immunoblotting. 5‐ALA/FAC treatment induced a rapid and transient increase in ERK phosphorylation, which peaked at 15–30 min and declined by 45 min, while AKT phosphorylation was similarly elevated (Fig. 3B).

Previous studies have shown that the combination of 5‐ALA and iron ions induces heme oxygenase‐1 (HO‐1) expression through the MAPK pathway in several cell types [19, 20, 21]. To determine whether this mechanism is also relevant in dermal papilla cells, we measured the expression of the HO‐1 gene (*HMOX1*) following 5‐ALA/FAC treatment. Quantitative PCR analysis demonstrated no significant changes in *HMOX1* mRNA levels at either 8 or 24 h (Fig. 3C). Together, these results indicate that 5‐ALA/FAC activates ERK and AKT signaling in iDPCs independently of HO‐1 induction, highlighting a distinct signaling context from that described in other cell systems.

### 5‐ALA/FAC promotes hair follicle‐like structure formation and hair growth

**Fig. 4:**
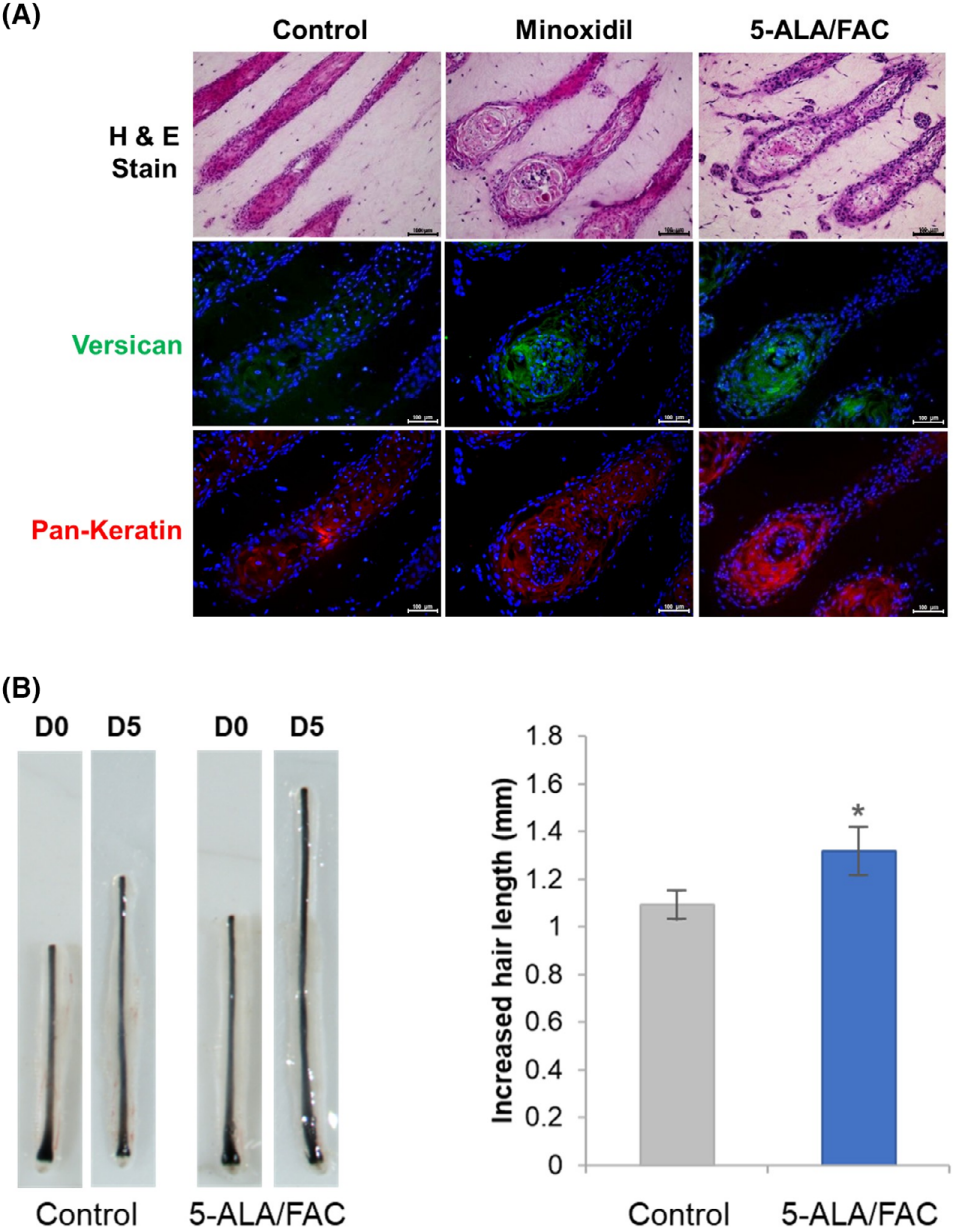
5‐ALA/FAC promotes hair‐follicle‐like structure formation and *ex vivo* hair‐shaft elongation. (A) Histological and immunofluorescence images of 3D reconstituted human skin (Neoderm®‐DF) treated with control, 100 μm minoxidil, or 0.6% 5‐ALA/FAC. H&E staining showed organized hair‐bulb and hair‐root structures. Versican (green) and pan‐keratin (red) immunostaining patterns were comparable between 5‐ALA/FAC‐ and minoxidil‐treated groups. Nuclei were counterstained with DAPI (blue). (B) *Ex vivo* human hair organ culture showing enhanced hair‐shaft elongation by 1 μm 5‐ALA/FAC for 5 days (D5). Representative images and quantitative analysis of hair length (mean ± SE, *n* = 20). Control samples were treated with vehicle only. *P* < 0.05 vs. vehicle control. Statistical analysis was performed using a two‐tailed unpaired Student's *t*‐test. Scale bars = 100 μm.

The ability of 5‐ALA/FAC to promote hair follicle formation was evaluated using a three‐dimensional (3D) reconstituted human skin model composed of DPCs and keratinocytes, which recapitulates the dermal papilla, hair bulb, and hair root structures [22]. The suitability of this system was first confirmed by analyzing the expression of structure‐specific proteins under control and internal positive control (minoxidil) conditions. In both control and minoxidil‐treated cultures, expression of pan‐keratin was detected in hair bulb and hair root structures. Compared with the control, minoxidil treatment resulted in the formation of a distinct hair bulb structure, characterized by densely aggregated cells within the bulb region, along with increased expression of versican. Notably, 5‐ALA/FAC treatment produced comparable changes to those observed with minoxidil, leading to the formation of a clearly defined hair bulb structure and enhanced versican expression (Fig. 4A).

To further evaluate the hair growth‐promoting effect of 5‐ALA/FAC, a human scalp hair follicle organ culture model was used. Hair follicles cultured with 5‐ALA/FAC for 5 days exhibited significantly greater elongation compared with control follicles, as shown by increased hair shaft length (Fig. 4B). These findings demonstrate that 5‐ALA/FAC not only induces hair follicle‐like structural organization in a 3D reconstituted human skin model but also directly promotes human hair growth in an *ex vivo* organ culture system.

## Discussion

In this study, we demonstrated that 5‐ALA/FAC enhanced the proliferation of hair follicle‐related cells, promoted the expression of hair‐inductive genes, and stimulated the formation of hair follicle‐like structures in a reconstituted human skin model. These findings indicate that 5‐ALA/FAC directly activates cellular pathways associated with the telogen‐anagen transition.

Historically, research on 5‐ALA has largely focused on its role as a mitochondrial metabolite and precursor in photodynamic therapy (PDT) [13]. Although several clinical observations have suggested that ALA‐PDT may induce hair growth [17], its underlying mechanism has remained uncertain because PDT generates reactive oxygen species (ROS) [23], which decrease the viability of target cells [24]. In fact, this cytotoxic property has been utilized for hair removal applications [25], and skin irritation has been noted as a potential side effect [26]. Based on evidence that iron limits protoporphyrin IX (PPIX) accumulation and thereby reduces ROS‐mediated cytotoxicity [27], a previous study topically applied 5‐ALA together with an iron salt without light activation and observed hair‐growth promotion in mice *in vivo* [18]. These findings suggested that 5‐ALA itself might possess intrinsic hair growth‐promoting activity. While that study was limited to mouse *in vivo* experiments and did not elucidate the underlying mechanism, our results from *in vitro* and *ex vivo* human follicle models provide direct evidence that 5‐ALA can stimulate hair growth‐related processes.

Versican, SOX2, and FGF2 are well‐established markers associated with the hair‐inductive function of dermal papilla cells (DPCs). Versican is highly expressed in DPCs during the anagen phase and nearly absent in the telogen phase [28]. SOX2 is specifically expressed in DPCs, and genetic ablation of SOX2 impairs hair shaft outgrowth through activation of BMP signaling [29, 30]. FGF2 is also expressed in DPCs and promotes their inductive activity via PDGF signaling [31, 32]. Collectively, these genes serve as functional indicators of DPC activation and the anagen‐promoting capacity of hair follicles. Therefore, agents that enhance the expression of such hair‐inductive genes may have the potential to regulate hair cycle progression and promote regeneration. In this study, 5‐ALA/FAC not only restored the DHT‐induced suppression of hair‐inductive genes, including *VCAN*, *SOX2*, and *FGF2*, but also increased their basal expression in the absence of stress conditions. Moreover, in the 3D reconstituted human skin model, 5‐ALA/FAC treatment induced the formation of hair bulb‐like structures containing densely aggregated DPCs with enhanced versican expression. These findings suggest that 5‐ALA/FAC has the potential to stimulate both hair maintenance and regeneration.

Taken together, this study demonstrates that 5‐ALA/FAC exerts biological effects on hair follicle‐related cells in *in vitro* and *ex vivo* human follicle models. While the present findings provide mechanistic insights into the cellular responses associated with hair follicle function, further studies, including *in vivo* and clinical investigations, are required to determine the relevance of these effects in the context of hair loss.

## Conflict of interest

Two of the authors (H.‐W.R. and S.K.) are affiliated with Easytem Co., Ltd.

## Author contributions

H.‐W.R., E.‐S.O., and S.K. were involved in conceptualization and methodology. H.‐W.R. and S.K. were involved in investigation, data curation, writing—original draft preparation. H.‐W.R., E.‐S.O., and S.K. were involved in writing—review and editing. S.K. was involved in supervision. All authors have read and agreed to the published version of the manuscript.

## Data Availability

The data that support the findings of this study are available from the corresponding author [S.K.] upon reasonable request.
